# The RGF/GLV/CLEL Family of Short Peptides Evolved Through Lineage-Specific Losses and Diversification and Yet Conserves Its Signaling Role Between Vascular Plants and Bryophytes

**DOI:** 10.3389/fpls.2021.703012

**Published:** 2021-07-20

**Authors:** Chihiro Furumizu, Shinichiro Sawa

**Affiliations:** Graduate School of Science and Technology, Kumamoto University, Kumamoto, Japan

**Keywords:** RGF/GLV/CLEL, signaling peptide, land plant evolution, 1KP project, *Marchantia polymorpha*, homology, convergence

## Abstract

Short secreted plant peptides act as key signaling molecules and control a plethora of developmental and physiological processes. The ROOT GROWTH FACTOR (RGF)/GOLVEN (GLV)/CLE-Like (CLEL) family of peptides was discovered to be involved in root development in *Arabidopsis thaliana*. In contrast to active research efforts, which have been revealing receptors and downstream signaling components, little attention has been paid to evolutionary processes that shaped the RGF signaling system as we know it in angiosperms today. As a first step toward understanding how RGF signaling emerged and evolved, this study aimed to elucidate the phylogenetic distribution and functional conservation of RGF-like sequences. Using publicly available, genome and transcriptome data, RGF-like sequences were searched in 27 liverworts, 22 mosses, 8 hornworts, 23 lycophytes, 23 ferns, 38 gymnosperms, and 8 angiosperms. This led to the identification of more than four hundreds of RGF-like sequences in all major extant land plant lineages except for hornworts. Sequence comparisons within and between taxonomic groups identified lineage-specific characters. Notably, one of the two major RGF subgroups, represented by *A. thaliana* RGF6/GLV1/CLEL6, was found only in vascular plants. This subgroup, therefore, likely emerged in a common ancestor of vascular plants after its divergence from bryophytes. In bryophytes, our results infer independent losses of RGF-like sequences in mosses and hornworts. On the other hand, a single, highly similar RGF-like sequence is conserved in liverworts, including *Marchantia polymorpha*, a genetically tractable model species. When constitutively expressed, the *M. polymorpha* RGF-like sequence (Mp*RGF*) affected plant development and growth both in *A. thaliana* and *M. polymorpha*. This suggests that MpRGF can exert known RGF-like effects and that Mp*RGF* is under transcriptional control so that its potent activities are precisely controlled. These data suggest that RGFs are conserved as signaling molecules in both vascular plants and bryophytes and that lineage-specific diversification has increased sequence variations of RGFs. All together, our findings form a basis for further studies into RGF peptides and their receptors, which will contribute to our understandings of how peptide signaling pathways evolve.

## Introduction

Debate is ongoing regarding phylogenetic relationships among land plant lineages. Gaining more support from recent molecular data, one hypothesis proposes that vascular and non-vascular plants are both monophyletic and stem from a deeply rooted split that took place early in land plant evolution (for schematic representation, refer to Figure 8). Morphologically, non-vascular plants (i.e., bryophytes) and angiosperms are quite different; homology at the organ level is not generally accepted or remains unresolved between the dominant gametophytic body of bryophytes and dominant sporophytic body of angiosperms. Nevertheless, recent molecular genetics studies have revealed cases where homologous genetic modules control development of functionally analogous organs in the two bodies of different lineages and generations (Koi et al., 2016; Rovekamp et al., 2016; Whitewoods et al., 2018; Yasui et al., 2019; Hirakawa et al., 2020). These observations could be accounted for by a common origin of organs in study or independent recruitment of the same genetic module to analogous organs. Either way, knowledge of molecular bases that underlie biological processes in diverse species is instrumental in understanding genetic mechanisms of how plants evolve and diversify.

Since the first discovery of an endogenous short signaling peptide, genetic, biochemical, and bioinformatic approaches have continued to add new signaling peptide families that act in various biological processes (Olsson et al., 2019; Segonzac and Monaghan, 2019). They mature from short precursor proteins through post-translational modifications and proteolytic cleavage into peptides of typically 10-to-20 amino acid residues (post-translationally modified peptides; PTMPs for short). More than ten PTMP families have been reported to date and all share common gene structures; PTMPs are encoded near the N-terminus. Outside the short PTMP-encoding region, PTMP precursor sequences are highly variable among family members. This structural feature has been hampering confident identification of PTMP homologs especially between two distantly related species. Nonetheless, studies started to reveal conservation of PTMPs across land plants (e.g., Miwa et al., 2009; Ghorbani et al., 2015; Tavormina et al., 2015; Goad et al., 2017).

ROOT GROWTH FACTOR (RGF)/GOLVEN (GLV)/CLE-Like (CLEL) is one of the widely conserved PTMP families (Shinohara, 2021). The RGF/GLV/CLEL (hereafter referred to as RGF for clarity) peptides were discovered independently in *Arabidopsis thaliana* (Matsuzaki et al., 2010; Meng et al., 2012; Whitford et al., 2012). Followed by the identification of their receptors, RGF1-INSENSITIVE (RGI)/RGF1 RECEPTOR (RGFR), the RGF-RGI module has been studied intensively, which in a short period of time has led to the independent elucidation of the downstream events after the receptor activation in *A. thaliana* (Ou et al., 2016; Shinohara et al., 2016; Song et al., 2016; Fernandez et al., 2020; Lu et al., 2020; Shao et al., 2020; Yamada et al., 2020). Gain-of-function alleles and exogenous application of RGF result in developmental defects in the root, and previous studies primarily focus on the roles of RGFs in root development. It has been noted from early on, however, that the expression of the RGF family genes is not restricted to the root (Ghorbani et al., 2016; Busatto et al., 2017). Likewise, although the RGF-RGI module has been studied so far only in angiosperms, bioinformatics analyses identified RGF homologs also in gymnosperm and lycophyte lineages of vascular plants (Strabala et al., 2014; Ghorbani et al., 2015). These limit our view on how this peptide signaling pathway evolved to play their roles as known today. Meanwhile, increasing availability of complete genome sequences enabled larger-scale bioinformatics analyses and led to the identification of RGF homolog in another vascular plant lineage, ferns (Furumizu et al., 2021). An RGF-like sequence was also found in a non-vascular plant, *Marchantia polymorpha* (designated as MpRGF for ROOT GROWTH FACTOR/GOLVEN/CLE-Like-FLAVORED) (Furumizu et al., 2021). These discoveries open up opportunities for studying RGF signaling pathways in different developmental or evolutionary settings. Yet, limiting searches to completely sequenced genomes excludes the possibility to fully consider the large diversity of land plants; a better understanding of the evolution of RGFs comes with a more comprehensive investigation among phylogenetically diverse species.

This study, therefore, searched for RGF-like sequences extensively in all major extant land plant lineages using a large-scale transcriptome data collected by the 1000 Plant Genomes Project (1KP^1^) as well as other valuable genome and transcriptome resources (Rensing et al., 2008; Banks et al., 2011; Goodstein et al., 2012; Amborella Genome Project, 2013; Fernandez-Pozo et al., 2015; Ming et al., 2015; Cheng et al., 2017; Filiault et al., 2018; Dong et al., 2019; One Thousand Plant Transcriptomes Initiative, 2019; Hetherington et al., 2020; Li et al., 2020; Zhang J. et al., 2020; Zhang L. et al., 2020; Geng et al., 2021). As a result, more than four hundreds of RGF-like sequences were retrieved from all major extant land plant lineages excepting hornworts. Mapping these RGF-like sequences on the species phylogeny revealed independent losses in mosses and hornworts and evolution of lineage-specific variations in vascular plants. We further demonstrated that MpRGF possesses known RGF-like activities and can affect plant growth with constitutive expression experiments in *A. thaliana* and *M. polymorpha*. This suggests that a role as a signaling molecule is conserved between vascular plant and bryophyte RGF-like sequences. Our findings form a basis for advancing studies into RGF peptides and their receptors and contribute to our understandings of how peptide signaling pathways evolve.

## Materials and Methods

### Identification of RGF-Like Sequences

Initial BLAST (Basic Local Alignment Search Tool) queries were assembled based on the previously published RGF-like sequences (Furumizu et al., 2021) and used to set up BLASTP or tBLASTn searches. The finalized query file is available as Supplementary Data Sheet 1. Fasta files of the published transcriptome and proteome data were downloaded to set up databases for BLAST searches. BLASTP was used to search against the proteome with the following parameters: -task blastp-short -max_target_seqs 30. tBLASTn was used to search against the transcriptome data with the parameters as follows: -word_size 2 -gapopen 9 -gapextend 1 -matrix PAM30 -threshold 16 -comp_based_stats 0 -window_size 15 -max_target_seqs 30. BLAST results were manually examined, and RGF-like candidates were listed. These hits were assessed by the sequence conservation of the presumptive mature peptides, which we defined to begin with the first aspartic acid and second tyrosine residues. When a potential full-length precursor sequence was available, the location of the mature peptide-encoding region and the presence of signal peptide were examined. Given that the processing and secretion modes of RGF-like peptides have not yet been elucidated outside of angiosperms, predicted presence of signal peptide was not considered as prerequisite. All amino acid sequences were aligned with the online version of MAFFT version 7 (Katoh et al., 2019) and manually edited and visualized with Jalview version 2.11.1.3 (Waterhouse et al., 2009). WebLogo version 2.8.2^2^ was used to generate sequence logos (Crooks et al., 2004) with the same clustalw color scheme used in Jalview. RGF-like sequences presented in this study are available in Supplementary Data Sheet 2.

### Plant Materials and Growth Conditions

*A. thaliana* Col-0 and *M. polymorpha* Tak-1 (Ishizaki et al., 2008) were used as wild type. Plants were grown at 23°C under continuous light. *A. thaliana* seeds were surface-sterilized and germinated on 140 mm × 100 mm × 14.5 mm square dishes (sterile No. 2 square Schale, Eiken Chemical) containing half-strength Murashige and Skoog (MS) Basal Medium (M5519, Sigma-Aldrich) solidified with 1% (w/v) agar (01028-85, Nacalai Tesque). The pH of the medium was adjusted to pH 5.7 with potassium hydroxide. Transformants were selected on half-strength MS plates containing 15 μg/mL glufosinate ammonium (079-05371, FUJIFILM Wako Pure Chemical) and 12.5 μg/mL carbenicillin sodium salt (037-23693, FUJIFILM Wako Pure Chemical). Plates were placed vertically during plant cultivation. For root observation, 1-week-old seedlings were transferred to the half-strength MS medium containing 1.0% sucrose (190-00013, FUJIFILM Wako Pure Chemical) and 1.8% agar, with (for transformants) or without (for Col-0) 15 μg/mL glufosinate ammonium and 12.5 μg/mL carbenicillin sodium salt. Plates were inclined at an angle of approximately 45° to the vertical. *M. polymorpha* plants were grown on 90 mm × 20 mm Petri dishes (BIO-BIK I-90-20, Ina⋅Optica) containing half-strength Gamborg’s B5 Medium (399-00621, FUJIFILM Wako Pure Chemical) solidified with 1.4% (w/v) agar. The pH of the medium was adjusted to pH 5.5 with potassium hydroxide. Transformants were selected on half-strength Gamborg’s B5 plates containing 10 μg/mL hygromycin B (085-06153, FUJIFILM Wako Pure Chemical) and 100 μg/mL cefotaxime sodium salt (030-16113, FUJIFILM Wako Pure Chemical). All nutrient agar plates were double wrapped with surgical tape (Micropore Surgical Tape 1530-0, 3M) during plant cultivation. Plant images were obtained by scanning plates using a flat-bed scanner (GT-X830, Epson).

### Construction of Plasmids

For constitutive expression in *A. thaliana*, the At*RGF1*, At*RGF6*, and Mp*RGF* coding sequences were PCR amplified from the Col-0 root-derived complementary DNA (cDNA), Col-0 genomic DNA, and Tak-1 gemmae-derived cDNA, respectively, cloned into the pART7 plasmid digested with *Bam*HI and *Xho*I using the In-Fusion HD cloning kit (639649, Takara Bio), and sequenced. The pART7 vector contains the cauliflower mosaic virus 35S promoter sequence and the terminator sequence from the octopine synthase gene. A 15-bp deletion was introduced into pART7 carrying Mp*RGF* by inverse PCR and confirmed by sequencing. Subsequently, the *Not*I fragment of the obtained plasmid was cloned into the *Not*I site of the pMLBART binary vector using the DNA ligation kit, mighty mix (6023, Takara Bio). For constitutive expression in *M. polymorpha*, the Mp*RGF* coding sequence was PCR amplified from the Tak-1 gemmae-derived cDNA, cloned into the pENTR4 dual selection vector (Invitrogen A10561, Thermo Fisher Scientific) digested with *Bam*HI and *Xho*I using the In-Fusion HD cloning kit, and sequenced. Subsequently, the Mp*RGF* coding sequence was subcloned into pMpGWB103 (Ishizaki et al., 2015) using the Gateway LR clonase II enzyme mix (Invitrogen 11791-100, Thermo Fisher Scientific). Sequences of the primers used in this study are listed in Supplementary Table 1.

### Plant Transformation

Constructs were introduced into the *Agrobacterium tumefaciens* strain GV3101 (pMP90) by electroporation to be used for plant transformation. *A. thaliana* plants were transformed by the floral dip method using surfactant Silwet L-77 (BMS-SL7755, Bio Medical Sciences) (Clough and Bent, 1998). *M. polymorpha* gemmalings were transformed as previously described (Kubota et al., 2013).

## Results

### RGF-Like Sequences Are Highly Conserved in Liverworts

**FIGURE 1:**
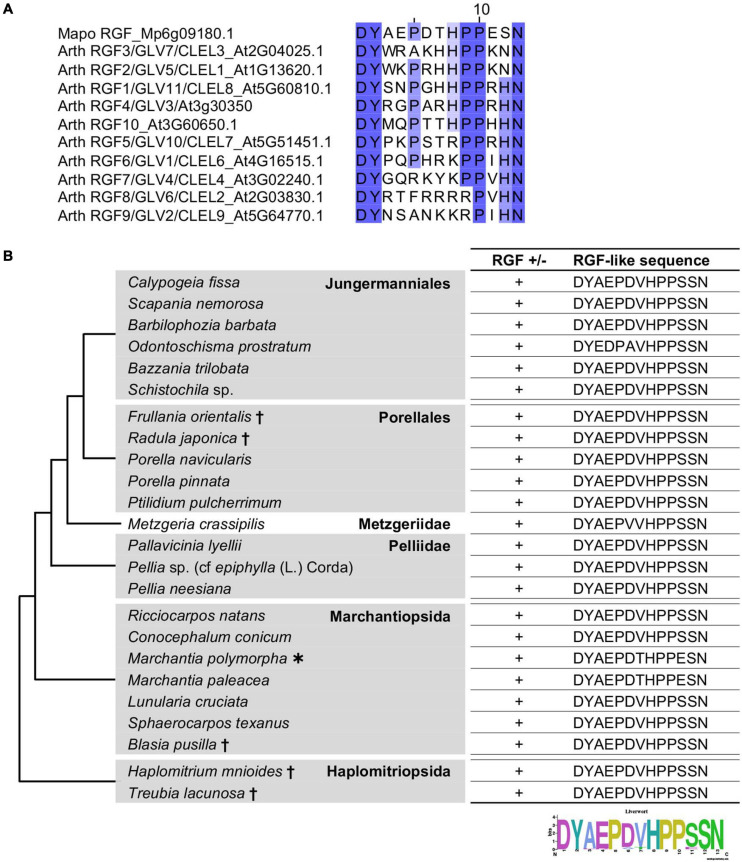
Liverwort RGF-like sequences. **(A)** Alignment of the predicted mature RGF peptides encoded in the *A. thaliana* and *M. polymorpha* genomes. Conserved residues are highlighted with graded shadings. Sequence titles are preceded by the abbreviated species name consisting of the first two letters of the generic and specific names. The sequence of *A. thaliana* RGF4 is based on a previous study (Matsuzaki et al., 2010). **(B)** Shown are the RGF-like peptide sequences identified in the selected liverworts. Phylogenetic relationships of the analyzed species are depicted as a simplified cladogram based on the published phylogenetic analyses (Forrest et al., 2006; Dong et al., 2019). A heavy asterisk indicates that the proteome predicted from the sequenced genome was analyzed for *M. polymorpha* (Bowman et al., 2017). Daggers indicate that the published transcriptome data was analyzed for these species (Dong et al., 2019). The 1KP dataset was analyzed for other species. See also Supplementary Figure 1.

Mature RGF peptides in *A. thaliana* (hereafter, AtRGFs) share several characteristic residues: the first aspartic acid (D), the second tyrosine (Y) to be sulfated, the tenth proline (P), and the thirteenth asparagine (N). The fifth and ninth P as well as the eighth histidine (H) residues are also highly conserved (Figure 1A). All these residues are conserved in the RGF-like sequence found in a liverwort, *M. polymorpha*, MpRGF (Furumizu et al., 2021; Figure 1A). A notable difference is the twelfth residue, which is either H or N in AtRGFs but is serine (S) in MpRGF, pointing to RGF sequence diversification between two evolutionary distant lineages. This prompted us to further explore the presence and sequence of putative RGFs in other liverworts.

The liverwort transcriptomes in the 1KP dataset was searched for RGF-like sequences. The 16 amino-acid sequences, encompassing the predicted mature peptide and three preceding residues, of AtRGFs and previously identified RGF-like sequences were used as query in the BLAST (Basic Local Alignment Search Tool) searches against the transcriptome data, translated in all six frames. This identified RGF-like sequences in 18 transcriptomes (Figure 1B). These findings corroborate the utility of the 1KP dataset in searching signaling peptide-encoding transcripts, which are often expressed at low levels. In the alignment of these sequences, the conservation stretches beyond the presumptive peptide-encoding region (Supplementary Figure 1), validating that several partial sequences lacking the N-terminus are indeed homologous to RGFs. In addition, the alignment identified two potential proteolytic cleavage sites in the variable, middle region of nascent polypeptides (Supplementary Figure 1). Equivalent residues are targeted by subtilisin-like serine proteases, and these processing events are crucial for the AtRGF biogenesis (Ghorbani et al., 2016; Stuhrwohldt et al., 2020). This suggests that liverwort RGF-like sequences can be processed to PTMPs by molecular machineries shared between vascular plants and bryophytes.

The identified RGF-like sequences are nearly identical. No RGF-like sequences were found in *Radula lindenbergiana*, *Monoclea gottschei*, and *Blasia* species listed in the 1KP dataset. In order to examine the presence of liverwort RGFs more widely, additional BLAST searches were performed using the transcriptome data in an independent study (Dong et al., 2019). This led to the finding of RGF-like sequences in the following five species: *Frullania orientalis*, *Radula japonica*, *Blasia pusilla*, *Haplomitrium mnioides*, and *Treubia lacunosa* (Figure 1B). Given that highly similar RGF-like sequences were uniformly conserved across liverworts, the absence of significant hits in the BLAST searches of several 1KP datasets is likely explained by the lack or the loss of low-abundance transcripts in the transcriptome data.

### RGF-Like Sequences Were Lost Independently During Moss and Hornwort Evolution

The conservation of RGF between angiosperms and liverworts indicates that the ancestral *RGF* gene(s) should have been present in the common ancestor of vascular plants and bryophytes (liverworts, mosses, and hornworts) while RGF-like sequences were not identified in the sequenced genomes of a moss, *Physcomitrium patens*, and *Anthoceros* hornworts (Rensing et al., 2008; Li et al., 2020; Zhang J. et al., 2020; Furumizu et al., 2021). Therefore, we investigated the possibility of finding moss or hornwort RGFs in the 1KP dataset.

**FIGURE 2:**
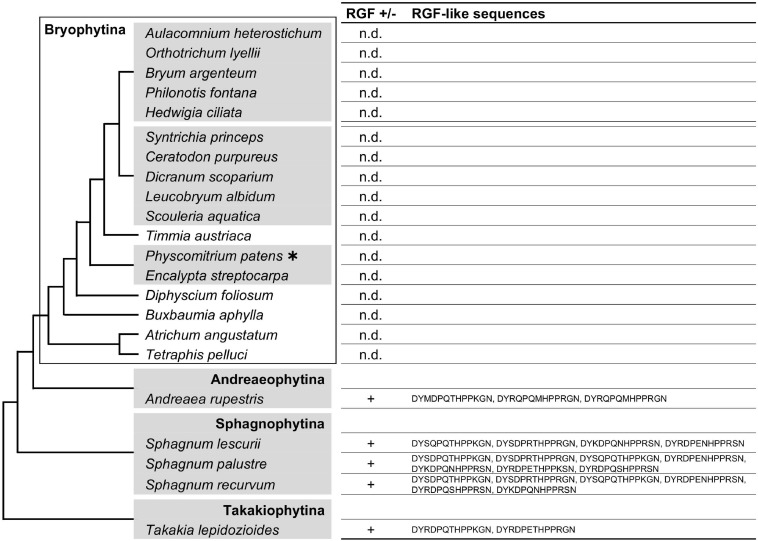
Moss RGF-like sequences. The presence (+) or absence (n.d., not detected) of RGF-like sequences was examined in mosses, and the identified, presumptive RGF-like peptide sequences are listed. Phylogenetic relationships of the analyzed species are depicted as a simplified cladogram based on the published phylogenetic analyses (Liu et al., 2019). A heavy asterisk indicates that the proteome predicted from the sequenced genome was analyzed for *P. patens* (Rensing et al., 2008). The 1KP dataset was analyzed for other species. See also Supplementary Figure 2.

RGF-like sequences were found in several but not all moss transcriptomes used in our searches (Figure 2 and Supplementary Figure 2). In order to examine these findings in the evolutionary context, the presence or absence as well as the identified sequences were mapped on the moss species phylogeny. This revealed that the RGF distribution is limited to the species that diverged early during the moss evolution (Figure 2). RGF-like sequences were not found in the species that belong to evolutionary younger lineages, collectively known as Bryophytina, suggesting that the RGF-like sequences were lost before the divergence of Bryophytina from Andreaeophytina. This is consistent with the previous report that RGF-like sequences are absent in the sequenced genome of *P. patens*, which belongs to Bryophytina. Alternatively, evolutionary changes in the expression pattern could also account for the lack of RGF-like sequences in Bryophytina in our results, through affecting the amount of RGF transcripts in a given transcriptome.

**FIGURE 3:**
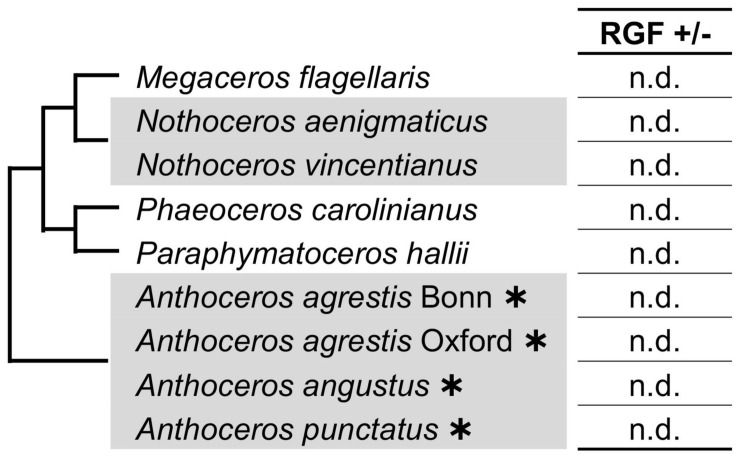
RGF-like sequences were not found in hornworts. Phylogenetic relationships of the analyzed species are depicted as a simplified cladogram based on the published phylogenetic analyses (Frangedakis et al., 2020). Heavy asterisks indicate that the proteome data predicted from the completely sequenced genomes were analyzed for the *Anthoceros* species (Li et al., 2020; Zhang J. et al., 2020). The 1KP dataset was examined for other species.

Searches in the hornwort transcriptomes did not find any RGF-like sequences. Taken together with the previous reports, in which RGF was not identified in the sequenced four genomes of *Anthoceros* species (Li et al., 2020; Zhang J. et al., 2020; Furumizu et al., 2021), these data suggest that during the hornwort evolution, RGF was possibly lost before extant lineages diversified (Figure 3). It is still yet possible that the low to zero abundance in the transcriptome data did not allow us to identify hornwort RGFs in this study.

### RGF-Like Sequences Diversified in Lycophytes

**FIGURE 4:**
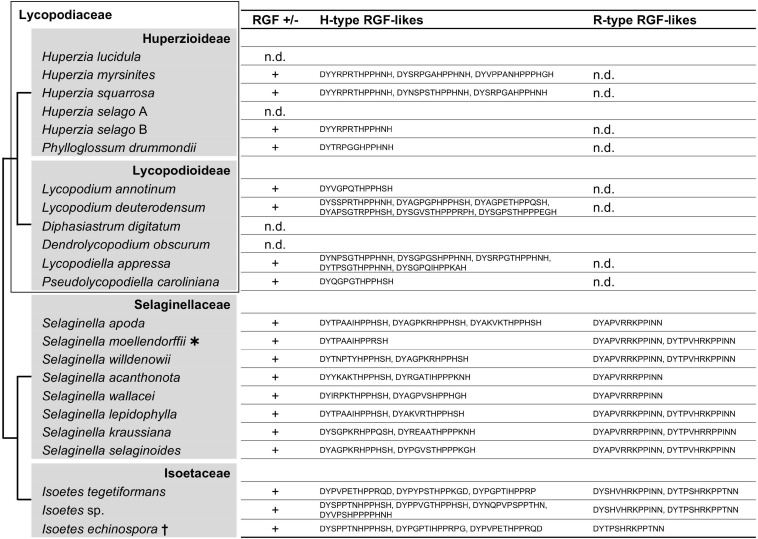
Lycophyte RGF-like sequences. The presence (+) or absence (n.d., not detected) of RGF-like sequences was examined in lycophytes. The identified, presumptive RGF-like peptide sequences are classified into two groups and listed. Phylogenetic relationships of the analyzed species are depicted as a simplified cladogram based on the published phylogenetic analyses (Wikstrom and Kenrick, 2000; Field et al., 2016; Weststrand and Korall, 2016; Schafran et al., 2018). A heavy asterisk indicates that the proteome predicted from the sequenced genome was analyzed for *S. moellendorffii* (Banks et al., 2011). A dagger indicates that the published transcriptome data was examined for *Isoetes echinospora* (Hetherington et al., 2020). The 1KP dataset was analyzed for other species. See also Supplementary Figure 3.

The C terminus of the liverwort and moss RGF-like sequences contains SSN and R/K-G/S-N, respectively (Figures 1, 2). These sequence features were not previously recognized for seed plant RGFs and can be explained by lineage-specific gene family evolution (Strabala et al., 2014; Ghorbani et al., 2015). Thus, we examined non-seed vascular plant lineages for hitherto unidentified sequence diversity. Lycophyte is a sister group to the ferns and seed plants and represents the earliest-diverging lineage of extant vascular plants (Spencer et al., 2020). *Selaginella moellendorffii* is the only lycophyte species of which genome was sequenced, and previous studies reported that RGF-like sequences are encoded in its genome (Banks et al., 2011; Ghorbani et al., 2015). To test other lycophyte species, lycophyte transcriptomes were selected from the 1KP dataset so that the large phylogenetic diversity of this lineage was captured and searched for RGF-like sequences. This led to the identification of RGF-like sequences from 17 out of 21 transcriptome data. Additional RGF-like sequences were retrieved by searching against the predicted *Selaginella moellendorffii* proteome and the *Isoetes echinospora* transcriptome data (Banks et al., 2011; Hetherington et al., 2020; Figure 4 and Supplementary Figure 3). No RGF-like sequences were found in four species of Lycopodiaceae (Figure 4). In the case of *Huperzia selago* (designated as A in Figure 4), a single RGF-like sequence was found in another transcriptome (B). This example suggests that the low transcript level can possibly hinder RGF identification in the 1KP dataset.

The lycophyte RGF-like sequences are classified into two major groups. Group H is characterized by the highly conserved eighth H residue in the predicted mature peptide-encoding region. The last H residue is also conserved with exceptions being five sequences found in the three *Isoetes* transcriptomes (marked with short black vertical line in Supplementary Figure 3). Unique residues at the C terminus differentiate these from more canonical Group H sequences. Besides, several Group H sequences have three P residues instead of the otherwise highly conserved two consecutive P residues at the ninth and tenth positions and could generate atypical 14 amino acid-long RGF peptides. The other, Group R, is characterized by the central R/K residues and the invariable twelfth and thirteenth N residues.

Differences between Group H and R are also found in the sequences adjacent to the predicted signaling peptide-encoding region. Most Group H sequences end with the predicted peptide-encoding region whereas additional 9-to-18 residues follow the peptide-encoding region of Group R, suggesting different modes of peptide maturation processes. These distinctive features indicate that two groups underwent significant sequence diversification if they share the same origin and thus are homologous. Pertinent to this is the presence of Group R sequences being limited to Selaginellaceae and Isoetaceae. This observation raises two possibilities: gain of Group R in the lineage leading to Selaginellaceae and Isoetaceae after its split from Lycopodiaceae (Huperzioideae and Lycopodioideae); and loss of Group R in the common ancestor of Lycopodiaceae. It is of note that *A. thaliana* RGFs can also be classified into Group H and Group R (Figure 1A). These will be discussed later in conjunction with our findings in other vascular plant lineages.

### Fern RGF-Like Sequences All Belong to Group H

The living sister group to lycophytes, euphyllophytes, consists of two major groups: seed plants and ferns. Ferns diverged from the seed plants 400 million years ago, and extant ferns are highly diverse (Plackett et al., 2015). Their early emergence and present-day diversity, however, has not always been sufficiently considered in previous phylogenetic studies, let alone in the studies of plant peptide signaling. It was recently reported that RGF-like sequences are encoded in the sequenced genomes of *Azolla filiculoides* and *Salvinia cucullata* (Furumizu et al., 2021). These are all similar to the Group H sequences in lycophytes, and it was not clear whether ferns have Group R-type sequences. We addressed this question by searching for RGF-like sequences in the selected fern transcriptomes of the 1 KP project as well as in the *Ceratopteris richardii* transcriptome reported independently (Geng et al., 2021).

**FIGURE 5:**
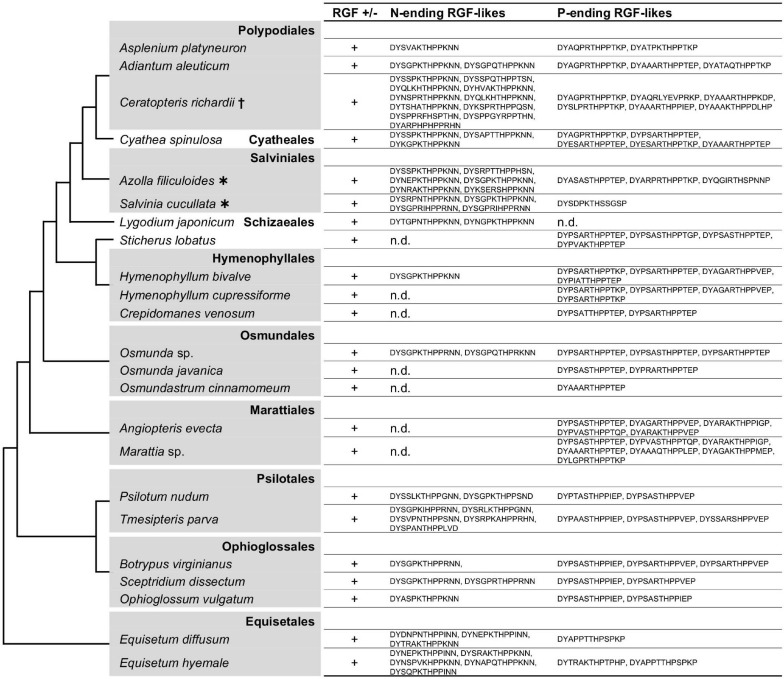
Fern RGF-like sequences. The presence (+) or absence (n.d., not detected) of RGF-like sequences was examined in ferns. The identified, presumptive RGF-like peptide sequences are classified into two groups and listed. Phylogenetic relationships of the analyzed species are depicted as a simplified cladogram based on the published phylogenetic analyses (Pryer et al., 2004; Knie et al., 2015; Schuettpelz et al., 2016). Heavy asterisks indicate that the proteome data predicted from the completely sequenced genomes were analyzed (Li et al., 2018). A dagger indicates that the published transcriptome data was examined for *Ceratopteris richardii* (Geng et al., 2021). The 1KP dataset was analyzed for other species. See also Supplementary Figure 4.

As shown in Figure 5 and Supplementary Figure 4, nearly all fern RGF-like sequences are characterized by the eighth H residue in the predicted mature form. No sequence was found to carry consecutive R/K residues at the seventh and eighth positions as seen in the lycophyte Group R sequences. The fern RGF-like sequences are classified into two groups according to the last residue of the predicted mature form: N in Group H-N, and P in the other, Group H-P (Supplementary Figure 4). These findings were mapped onto the fern species phylogeny (Figure 5). Either Group H-N or H-P sequences are absent in several fern species. In particular, Group H-N sequences were not found in *Angiopteris evecta* and *Marattia* sp., both of which belong to Marattiales. Group H-N may have been lost in this lineage, and validation of this possibility requires more species sampling.

### Gymnosperms Have Both Group H and Group R RGF-Like Sequences

The lack of Group R sequences in ferns and its presence in lycophytes and angiosperms obscures the evolutionary history of Group R RGF-like sequences in vascular plants. To gain an insight into how Group R sequences emerged and evolved, RGF-like sequences were analyzed in gymnosperms that represent an informative node, bridging seed-free vascular plants and angiosperms. Gymnosperms comprise only 1,000 species but consist of a wide variety of groups. Species were therefore sampled broadly from the available 1 KP dataset.

**FIGURE 6:**
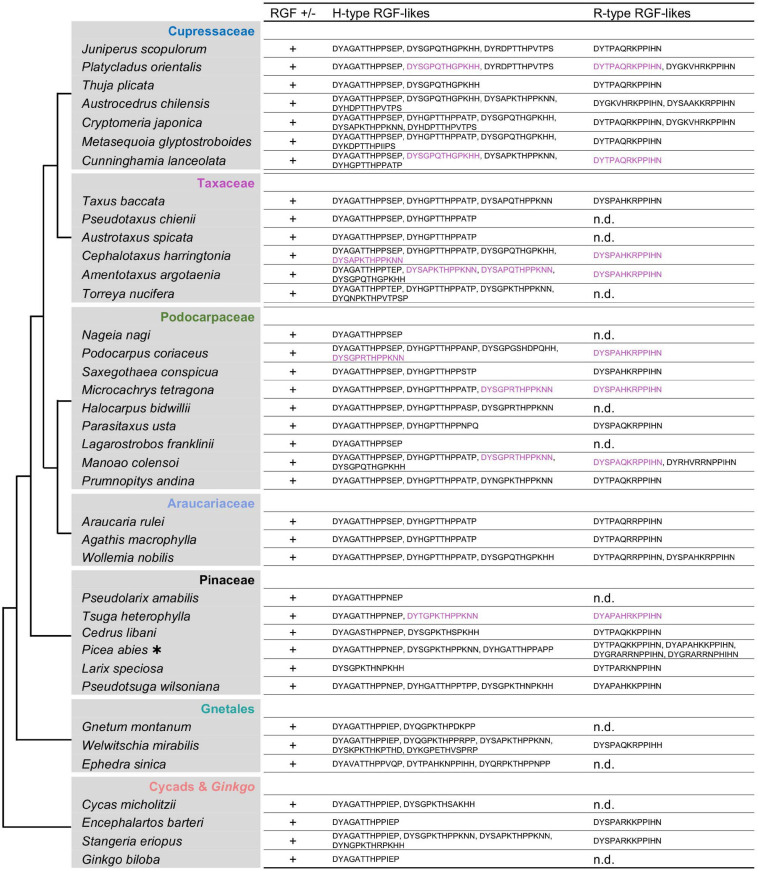
Gymnosperm RGF-like sequences. The presumptive RGF-like peptide sequences identified in gymnosperms are classified into two groups and listed. Sequences in magenta in a given species share similar N-terminal sequences, suggesting the common origin. R-type sequences were not found in several species (n.d., not detected). Phylogenetic relationships of the analyzed species are depicted as a simplified cladogram based on the published phylogenetic analyses (Lu et al., 2014; Ran et al., 2018; One Thousand Plant Transcriptomes Initiative, 2019). Names of taxonomic groups are color coded consistently with Supplementary Figure 5. A heavy asterisk indicates that the proteome predicted from the genome sequence was analyzed (Nystedt et al., 2013). The 1KP dataset was analyzed for other species. See also Supplementary Figures 5, 6.

Collected gymnosperm RGF-like sequences include both Group H and Group R sequences (Figure 6 and Supplementary Figure S5). This is consistent with a previous study, which also identified RGF-like genes of both types in coniferous species (Strabala et al., 2014). Several transcriptomes lack Group R sequences. The corresponding species do not belong to specific taxonomic groups, and Group R includes sequences from all major taxonomic lineages. This suggests that lower expression levels but not their absence in the genome accounts for the lack of Group R sequences in these transcriptomes. In fact, the sequenced *Picea abies* genome encodes seven RGF-like sequences in total, four of which belong to Group R. A mere number of zero-to-two in other species appears consistent with the hypothesis of lower transcript accumulation levels of Group R sequences in general.

Gymnosperm Group H sequences were further classified into five subgroups (Supplementary Figure 5). Subgroup H1 sequences were found only in Cupressaceae and Taxaceae, possibly having originated recently in the common ancestor of these evolutionary young lineages. Similar to Group R, other subgroups are more uniformly distributed in gymnosperms. Exceptions to this are subgroup H3 lacking cycad and Ginkgo sequences and subgroup H5 without Araucariaceae sequences. Given the lack of completely sequenced genomes in these lineages and the large size of gymnosperm genomes, it requires additional investigation to confirm lineage-specific sequence variations. Group H as a whole comprises sequences from all major gymnosperm lineages. Therefore, both Group H and R sequences must have been present in the common ancestor of extant gymnosperm species.

Manual curation of gymnosperm RGF-like precursor sequences revealed that several group H and R sequences show striking similarities (Supplementary Figures 5 and 6). These paired sequences were recognized so far only in several gymnosperm lineages in our searches and could possibly result from alternative splicing as reported by Strabala et al. (2014). Cross comparison between paired examples of different taxonomic groups did not show sequence conservation beyond mature-peptide encoding regions. This suggests their possible independent origins and raises a question as to what drove their evolution. Further investigations are needed to elucidate how commonly these pairs exist both within and outside gymnosperms.

### Cryptic Sequence Diversity in Angiosperm RGFs

**FIGURE 7:**
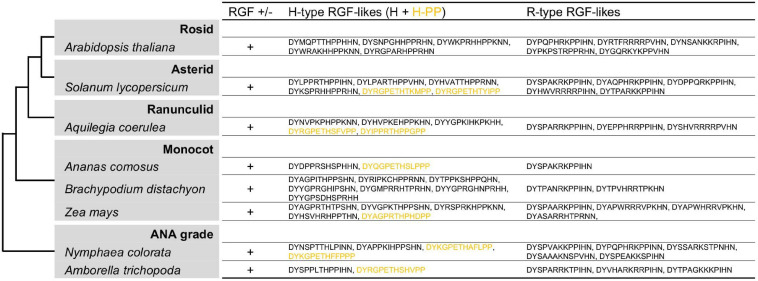
Angiosperm RGF-like sequences. The presumptive RGF-like peptide sequences identified in the sequenced genomes of angiosperms are classified into two groups and listed. Sequences in dull yellow belong to the group H-PP. Phylogenetic relationships of the analyzed species are depicted as a simplified cladogram based on the published phylogenetic analyses (One Thousand Plant Transcriptomes Initiative, 2019). See also Supplementary Figure 7.

RGF-like sequences were originally identified in *A. thaliana* and has since been reported in other angiosperm species (Matsuzaki et al., 2010; Meng et al., 2012; Whitford et al., 2012; Ghorbani et al., 2015; Tadiello et al., 2016). These angiosperm sequences, however, have not been characterized from an evolutionary perspective by comparing their profiles with those of RGF-like sequences from non-flowering plants. In view of the rapidly growing availability of plant genome data, RGF-like sequences were searched in diverse angiosperm species (Goodstein et al., 2012; Amborella Genome Project, 2013; Fernandez-Pozo et al., 2015; Ming et al., 2015; Cheng et al., 2017; Filiault et al., 2018; Zhang L. et al., 2020; Figure 7 and Supplementary Figure 7). This resulted in identifying both Group H and R sequences in all species examined. In addition, similar but distinct sequences were found to constitute a new subclass of Group H. Their presumptive mature forms in 13 amino acids end with PP and are hereby named Group H-PP. Some Group H-PP sequences have a C-terminal extension longer than 20 amino acids. It remains to be tested whether they encode secreted short peptides.

### Conserved and Lineage-Specific Features of RGF-Like Sequences

**FIGURE 8:**
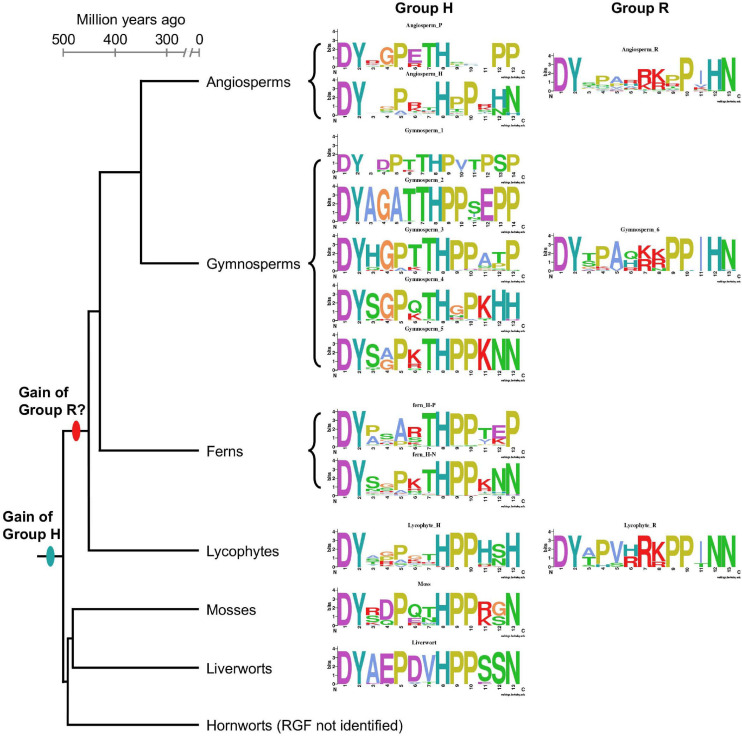
Lineage-specific features of RGF-like sequences. Sequence logos of the identified RGF-like sequences are shown along with a plausible phylogenetic tree of the extant, major land plant groups. The branch lengths are to the scale of the estimated divergence dates (Morris et al., 2018).

As summarized in Figure 8, RGF-like sequences show both conserved and lineage-specific characteristics. Group H sequences are found in all major lineages of extant land plants, suggesting an early origin of Group H in the common ancestor of land plants. Group R sequences, on the other hand, are present only in vascular plants. It is possible Group R evolved early in vascular plant lineage and was subsequently lost in ferns. Alternatively, Group R sequences may have evolved independently at least twice in ancestral lycophytes and seed plants. The latter could explain the difference in the twelfth position of predicted, mature RGF-like peptides, which is dominated by N in lycophytes and H in seed plants. Other sequence characteristics are highly similar between seed plant and lycophyte Group R sequences, in favor of their common origin.

Compared with Group R, Group H sequences show more diversity. This is of particularly the case in vascular plants. It has to be emphasized that except for *A. thaliana* RGFs, secreted, bioactive forms have not been determined (Matsuzaki et al., 2010; Whitford et al., 2012). Such structural information will be critical to delve into the sequence diversity of RGF-like sequences.

### *M. polymorpha* RGF-Like Sequence Elicits RGF-Like Activities in *A. thaliana*

As a first step toward understanding biological functions of RGF-like sequences in non-flowering plants, the *M. polymorpha* RGF-like sequence was chosen for molecular experiments for the following reasons. First, liverworts including *M. polymorpha* and other bryophyte groups form a presumably monophyletic lineage sister to vascular plants and serve as an informative reference to complement our knowledge on angiosperm RGFs (Figure 8). Second, a single, highly similar RGF-like sequence is present in the liverwort species analyzed in this study (Figure 1). This observation points to a conserved role for liverwort RGF-like sequences. Third, canonical proteolytic cleavage sites are identified at similar positions in the liverwort and *A. thaliana* RGF precursors (Ghorbani et al., 2016; Stuhrwohldt et al., 2020; Supplementary Figure 1). This led us to reason that conserved molecular players or analogous mechanisms for the biogenesis of RGF-like peptides could exist in *M. polymorpha* and *A. thaliana*. Fourth, the predicted *M. polymorpha* RGF-like sequence has several residues not found in angiosperm RGFs (Figures 1, 7, 8). These variations could possibly affect ligand-receptor interactions, which can be tested by heterologous expression.

**FIGURE 9:**
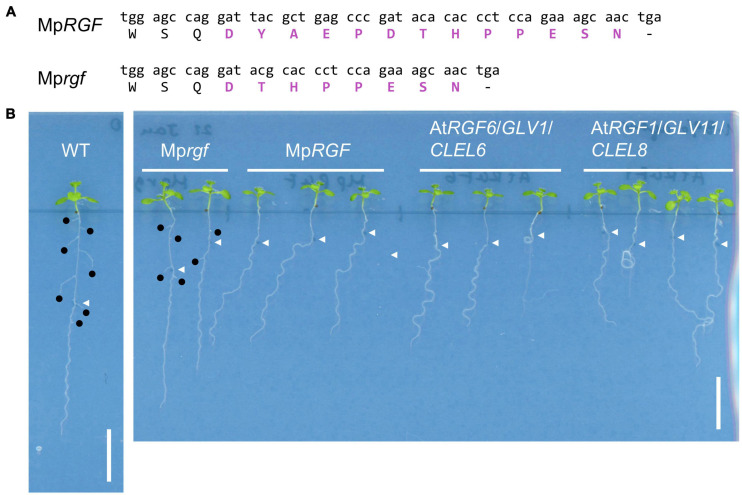
Overexpression of Mp*RGF* in *A. thaliana*. **(A)** A mutated form of Mp*RGF*, designated as Mp*rgf*, used in this experiment. Shown are the nucleotide and translated amino acid sequences encompassing the mature peptide encoding region. The deduced mature peptide sequences are highlighted in bold magenta letters. **(B)** Constitutive expression of At*RGF1*/*GLV11*/*CLEL8*, At*RGF6*/*GLV1*/*CLEL6*, Mp*RGF*, or its mutated form, Mp*rgf* in *A. thaliana* seedlings. A Col-0 plant is shown for comparison. Note the difference in plant vigor due to the chemicals added in the medium for selecting transgenic plants. White arrow heads indicate the position of the root tip at the time of seedling transfer to the hard agar medium plate. Emerged lateral roots are marked with black circles. Scale bars: 1 cm.

In *A. thaliana*, it has been reported that constitutive expression and exogenous application of RGFs affect root development (Matsuzaki et al., 2010; Meng et al., 2012; Whitford et al., 2012; Fernandez et al., 2013, 2015). We generated transgenic *A. thaliana* plants constitutively expressing either *A. thaliana* RGF (At*RGF1*/*GLV11*/*CLEL8* of Group H or At*RGF6*/*GLV1*/*CLEL6* of Group R) or *M. polymorpha* RGF (Mp*RGF*, Group H) and compared their root morphologies (Figure 9). In order to circumvent differences in the number and expression level of the transgene among independent lines, plants in the T1 generation were used. When grown on inclined, hard-agar plates, Mp*RGF*-expressing plants showed enhanced wavy growth. The overall direction of their root growth was bended. These phenotypes are similar to those caused by At*RGF1*/*GLV11*/*CLEL8* over-expression (Meng et al., 2012). At*RGF1*/*GLV11*/*CLEL8*-expressing plants formed irregular waves including loops as previously reported (Whitford et al., 2012). These morphologies are hardly observed in Mp*RGF*-expressing plants. Common to all three transgenic lines are the phenotypes such as shorter roots and lower density of lateral roots (Fernandez et al., 2013; Figure 9B). These phenotypes were not observed in *A. thaliana* plants constitutively expressing a mutated form of Mp*RGF*, of which predicted mature form lacks the first five amino acids (Mp*rgf*, Figure 9A). Therefore, the observed biological activities of MpRGF in *A. thaliana* are attributable to the putative peptide-encoding region. Despite notable sequence differences from angiosperm RGFs, our finding suggests that MpRGF can signal through the *A. thaliana* RGF receptors.

### Over-Expression of Mp*RGF* Results in Dwarfing in *M. polymorpha*

Our current understanding of the roles of RGFs is based on the previous studies focusing on the sporophytic organs of angiosperms, and it has not been reported to date that RGFs have any roles during gametophyte development. In contrast to angiosperms, in which the sporophyte generation is dominated, liverworts have a life cycle with the dominant gametophytic phase. It is thus more feasible to study RGF functions in the gametophyte using liverworts. Detection of RGF-like sequences in the 1KP dataset (Figure 1) corroborates that liverwort RGF-like sequences are expressed in their gametophytic bodies, from which samples are typically collected for transcriptome analyses (One Thousand Plant Transcriptomes Initiative, 2019). In *M. polymorpha*, a genetically tractable liverwort model (Bowman et al., 2017; Kohchi et al., 2021), publicly available RNA-Seq data shows that the Mp*RGF* transcript (Mp6g09180.1) is detected both in vegetative and reproductive organs with higher mRNA abundance in gametophytic tissues than in sporophytic tissues (MarpolBase^3^).

**FIGURE 10:**
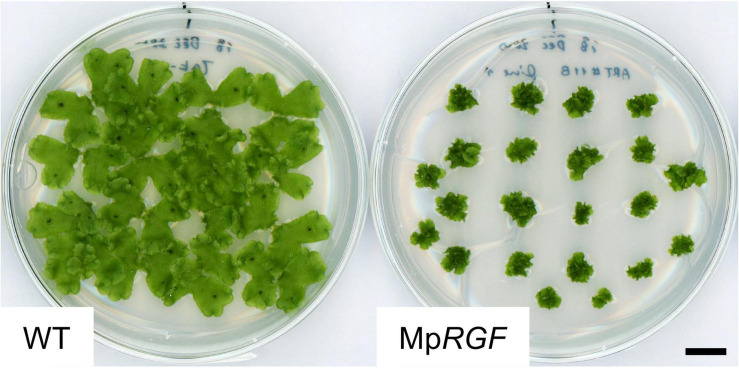
Overexpression of Mp*RGF* in *M. polymorpha*. *M. polymorpha* plants ectopically expressing the Mp*RGF* cDNA are shown with wild-type Tak-1 plants. G1 gemmalings plated simultaneously were observed. Scale bar: 1 cm.

To explore gametophytic RGF roles, the impact of activated RGF signaling was evaluated. For this purpose, transgenic M. *polymorpha* plants expressing Mp*RGF* under the constitutive Mp*EF1* promoter (Althoff et al., 2014) were generated by transforming regenerating thalli. Independent twelve T1 lines were distinguishable from wild-type plants by their small size. Gemma cup and gemmae formation was delayed but eventually observed in the Mp*RGF* overexpressors. This allowed us to grow wild-type and transgenic G1 gemmae simultaneously, which confirmed the observations in the T1 generation (Figure 10). That is, excessive MpRGF interfers with normal growth and development. We infer that Mp*RGF* is under strict transcriptional regulation so that its potent activities are controlled tightly.

## Discussion

### Framing Sequence Diversity of Signaling Peptides in the Context of Land Plant Evolution

Despite being as short as a chain of 10-20 amino acids, plant signaling peptides carry a large amount of information for binding with interacting partners to induce downstream signaling events. Much attention has been paid to understand the effect of peptide sequence variation on ligand-receptor interactions and resultant signaling responses within a few model species. These efforts have been contributing to an understanding of signaling peptide language, so to say, and even to the creation of a new one or a new word (peptide). The latter, for instance, was realized through a serendipitous discovery using a chemical synthetic approach (Hirakawa et al., 2017). These previous studies were driven by collecting, comparing, and clustering peptide sequences based on similarities. There exist, however, potential pitfalls associated with the common features found in signaling peptide encoding genes; namely, except for the short mature peptide encoding region, a large part of precursor proteins lack sequence conservation. This poses a challenge when identifying signaling peptides and analyzing their sequence variation with conventional homology-based and molecular phylogenetic approaches. As an attempt to overcome these shortcomings, we placed RGF-like peptides in a species phylogeny as summarized in Figure 8. This offers an opportunity to decipher evolutionary trajectories of RGF-like peptides and lay a foundation for further exploration of driving forces that underlie changes in peptide sequences.

### Challenges in Translating Bioinformatic Knowledge Into an Understanding of Living Systems

The unparalleled 1KP project as well as other valuable resources allowed us to identify more than four hundreds of RGF-like sequences in all major extant land plant lineages excepting hornworts. The absence of RGF-like sequences in our searches can result from the nature of the original transcriptome data, such as the lack or the loss of low-abundance transcripts in the tissue used for RNA extraction. Therefore, additional investigation by PCR-based homolog detection and ultimately by genome sequencing is necessary to confirm the lack of RGF-like sequences in a given species. This poses an intrinsic limitation to the transcriptome data. Moreover, a question remains unsolved as to whether these genes are all homologous due to the overall low sequence similarity. Homology itself is not a clear-cut concept (Inkpen and Doolittle, 2016), which one confronts when assessing similarities of sequences found in distantly related species. Pertinent to this is likely independent evolution of sequences similar to plant signaling peptides in animals and microbes (Mitchum et al., 2012; Ronald and Joe, 2018). To our knowledge, horizontal gene transfer has not been demonstrated in any of these cases of so-called peptide mimics, which can hijack the host peptide-receptor signaling pathways. A problem will thus linger of how to define a signaling peptide family, for instance, by homology or by interacting receptor.

Experimental validations are fundamental to confirm that the newly identified genes encode secreted short peptides and to determine amino acid sequences of their bioactive forms before postulating mechanisms that drive and generate a peptide sequence diversity. These biochemical analyses, however, are not always feasible in every species. We took an alternative and complementary approach, heterologous expression in *A. thaliana*, and demonstrated that the *M. polymorpha* RGF sequence shows RGF-like activities in plants (Figure 9). The result also showed that Mp*RGF*-induced responses are more similar to the phenotypes caused by constitutively expressing At*RGF6*/*GLV1*/*CLEL6* than At*RGF1*/*GLV11*/*CLEL8*, while MpRGF resembles the latter *A. thaliana* RGF in sequence (Figure 1). Such discrepancies at first glance can result from differences in expression levels or heterologous nature of the experiment; they could be reconciled in future studies if we gain a better understanding of ligand-receptor interactions both in *A. thaliana* and in *M. polymorpha*.

### Evolutionary Understanding Facilitates Mechanistic Studies in Model Species

In *A. thaliana*, multiple receptors have been reported for RGF peptides (Ou et al., 2016; Shinohara et al., 2016; Song et al., 2016). As pointed out previously, it remains unknown whether the RGF receptors have different affinities for specific RGF peptides *in vivo* (Pruitt et al., 2017). It was reported, however, that one of the *A. thaliana* RGF receptors (At4g26540) interacts with RGFs *in vitro* with different binding affinities (Song et al., 2016). Overall, H-type RGFs have higher affinities than R-type RGFs, suggestive of inherent differences among RGF ligand-receptor pairs in signaling outputs. Functional differences among RGFs have been noted since their discovery. For example, the H-type, and not R-type, acts as primary RGF in maintaining the meristematic activities in the *A. thaliana* root (Matsuzaki et al., 2010). Given that Group H RGF-like sequences are more widely conserved across land plants, with its emergence predating the split between vascular plants and bryophytes (Figure 8), when studying roles of RGFs in biological processes of interest, it can be helpful to keep in mind evolutionary differences between two major RGF subgroups; they show structural differences as well as changes in expression patterns. A recent study reported a novel role for R-type RGF, *A. thaliana* RGF7, in triggering innate immunity through interactions with a subset of RGF receptors (Wang et al., 2021). This opens up a new avenue to dissect how RGF signaling diversified.

### Mechanisms Underlying Functional Evolution of Peptide Ligand-Receptor Signaling

In the two examples of known RGF functions described above, the angiosperm root possibly evolved in the common ancestor of ferns and seed plants. Innate immunity in non-flowering plants is just beginning to be addressed (Ponce de León and Montesano, 2017; Carella et al., 2019; Matsui et al., 2020; Poveda, 2020). Therefore, it is not yet straightforward to transfer these knowledges in *A. thaliana* into studies on bryophyte and non-flowering vascular plant RGFs. In order to understand the evolution of RGF signaling, it is crucial to reveal biological roles of RGFs especially in the non-vascular plant lineage, bryophytes. Losses of RGF in hornworts and the moss lineage including *P. patens* directed our attention to the liverwort, *M. polymorpha*. Our results indicate that RGF-like sequences exist possibly as a single gene in liverworts. The high sequence similarities suggest their conserved roles in this lineage. The altered growth and development of Mp*RGF*-overexpressing plants indeed verifies its potency as a signaling molecule (Figure 10).

In future studies, it is of critical importance to identify MpRGF receptor(s). Homologs of *A. thaliana* RGF receptors are strong candidates as demonstrated for another signaling peptide family (Whitewoods et al., 2018; Hirakawa et al., 2019, 2020). In accordance, when expressed in *A. thaliana*, MpRGF appears to activate RGF receptor signaling (Figure 9). Assigning receptors to conserved signaling peptides in diverse lineages helps us dissect pairing rules of peptide ligands and receptors (Song et al., 2016; Zhang et al., 2016). The understanding of ligand-receptor interactions, in turn, provides insight into how they co-evolve.

Contrasting phylogenetically widespread RGFs, several signaling peptide families, such as LURE, PEP, and SCR/SP11, appear less conserved (Bartels and Boller, 2015; Nasrallah, 2017; Takeuchi, 2021). These presumably evolutionary-young families share intertwined features in common: rapid sequence diversification and participation in inter- or intra-species interactions with each influenced by the other (Takeuchi and Higashiyama, 2012; Lori et al., 2015; Ma et al., 2016; Murase et al., 2020). Understanding structural details of the evolving interface of homologous ligand-receptor pairs as well as underlying genetic changes has been advanced (Ma et al., 2016; Durand et al., 2020; Murase et al., 2020). Studies into RGF-like molecules and their receptors could offer opportunities to study ligands and receptors co-evolving over a longer period of time, from which related yet functionally distinct ligand-receptor pairs may emerge, for instance (van Kesteren et al., 1996).

In addition to ligand-receptor binding, other protein-protein interactions are also indispensable for functional peptide signaling and fine-tuning its activities; processing and modification enzymes involved in RGF peptide maturation have been studied in *A. thaliana* (Matsuzaki et al., 2010; Ghorbani et al., 2016; Stuhrwohldt et al., 2020). Assuming that the nascent MpRGF protein produces a short secreted peptide similar to mature AtRGFs, our finding supports that similar processing machineries are responsible for the MpRGF biogenesis (Supplementary Figure 1). Consequently, residues needed to interact with processing or modification enzymes should be conserved in the variable region of peptide precursors, where sequence similarities are seldom recognized, thereby constraining the primary and possibly higher structures of peptide precursors. Alternatively, changes in interactions can add new moieties to the signaling peptidome. These point to potential values of comparative evolutionary approaches in studying signaling peptide biogenesis.

Another key issue is to determine whether angiosperm and liverwort RGF signaling pathways involve any conserved cellular processes both in the sporophyte (dominant in angiosperms) and gametophyte (dominant in liverworts) generations (Fernandez et al., 2020; Lu et al., 2020; Shao et al., 2020; Yamada et al., 2020). Answers to this question will shed light on molecular mechanisms underlying diversification of land plants and, along with elucidation of RGF signaling components, will contribute to our understandings of how peptide signaling pathways evolve.

## Data Availability Statement

The datasets presented in this study can be found in online repositories. The names of the repository/repositories and accession number(s) can be found in the article/Supplementary Material.

## Author Contributions

CF conceived and designed the study and collected and analyzed the data. SS contributed to the materials. CF wrote the article with contributions from SS. Both authors contributed to the article and approved the submitted version.

## Conflict of Interest

The authors declare that the research was conducted in the absence of any commercial or financial relationships that could be construed as a potential conflict of interest.

## Footnotes

1 www.onekp.com

2 https://weblogo.berkeley.edu

3 https://marchantia.info
