# Supplementary material for: The RGF/GLV/CLEL Family of Short Peptides Evolved Through Lineage-Specific Losses and Diversification and Yet Conserves Its Signaling Role Between Vascular Plants and Bryophytes
Source: Front Plant Sci. 2021 Jul 20;12:703012. doi: 10.3389/fpls.2021.703012 (PMC8329595; doi:10.3389/fpls.2021.703012)
Supplement: Supplementary file 3 [file Data_Sheet_3.pdf]

Supplementary Material

```
                                                                                             60
Cafi RTMU-2183659            ----------------MVTGGRGDHAQPSAGCRIRCIHLV--LLLE--CLLFSIGTASPS
Scne IRBN-2167015            ----------------MSNGKSE--RGTSASWRARCMLLV--LLVQ--LSMLSIGTGSFS
Baba OFTV-2024263            ----------------MLNGNRG--GRTSARWRARCMLLV--SLLE--ISLISIGTASFS
Odpr YBQN-2008974            ----------------M-------------------------------------------
Batr WZYK-2090729            ----------------MVNGIRG--ARTSAGWRVRCMYLV--LLLE--FLLFAVGNATPS
Sc sp. LGOW-2014377          ----------------M-------------------------------------------
Fror SRR8202176_DN2013       -------------------------------------------------MLCTSFNARPV
Raja SRR8202175_DN137879     ---------------MSSRGGDA--KCGSARWRSRCIYLV--ILFE--FLSFPVGSADPS
Pona KRUQ-2018380            ----------------MLRGGSG--GCTSAKWQARCMCLV--LILE--FLQFSIGSAIPS
Popi UUHD-2013991            ----------------MLRGGSG--GCTSAKWRARCMCLI--FILE--FVQFSIGSAIPS
Ptpu HPXA-2014466            ------------------------------------------------------------
Mecr NRWZ-2048038            ------------------------------------------------------------
Paly YFGP-2105141            ------------------------------------------------------------
Pe sp. PIUF-2010895          ------------------------------------------------------------
Pene JHFI-2109909            ------------------------------------------------------------
Rina WJLO-2009253            ----------------MFRCKSDR---------ARYASLALLLLLN--LLFVTTGSATAF
Coco ILBQ-2045467            ----------------MFRCRS--------EW-ARCISLA--LLLQLNVLFITTGSAIPL
Mapo Mp6g09180.1             ----------------MFRCRS--------EW-ARCISLALLLLLK--LLFMTTGSAIPL
Mapa HMHL-2053619            ----------------MFRCRS--------EW-ARCISLALLLLLK--LLFMTTGSAIPL
Lucr TXVB-2075053            ----------------MFRGRS--------AW-TRCINLV--LLLDL-LLFMTCGSAIAL
Spte HERT-2044039            ----------------MHNSIAT----RKRAW-SRCIRLA--LLLD--LLFMTTGSAIPR
Blpu SRR8202193_DN28790      MYSLQGCIFPHLSILNMLRGGS--------EW-VRYICLA--LLLD--LLLISTGSAIPL
Hamn SRR8202204_DN3233       ----------------MYKSRL--------AW-SWCISFT--VVLQ--LLMICTGSPSAV
Trla SRR8202205_DN12243      ----------------MFKTRI--------AW-TRFISFT--VLLQ--LLLICTESTVDI

                                                                                            120
Cafi RTMU-2183659            -RKEIGDSVVSI---YNSLTAADK---VHNSLNFHP-ALQESEVYPAQQDSFFPEVSTD-
Scne IRBN-2167015            -NKGNVEQMSAV----DQSLTPGE---LRSVLNSYP-ALQEFEVHSTLKDSSFPGVVDS-
Baba OFTV-2024263            -NKENVDQKVAV----DQSSTPGK---LRSVLNSYP-ALQEFQVHPTQKDPSFPEVVGS-
Odpr YBQN-2008974            ------------------------------------------------------------
Batr WZYK-2090729            -RQEYGNDPMAPT-VDDSTTPATK---LRNTLNSYP-ALREFEVHSTRKGAIFPEVGW--
Sc sp. LGOW-2014377          ------------------------------------------------------------
Fror SRR8202176_DN2013       ------------------------------------------------------------
Raja SRR8202175_DN137879     -NKESTHNLGTV---SGSAIPPTD---LRGQSHDHSRPPSGVHVYVLSKVNQMPKSQ---
Pona KRUQ-2018380            -TKGYGAQVAPA---DDSSIAATR---LRSY-SNYC-PASLLEVHSKPKGDLFPDVILKS
Popi UUHD-2013991            -TKGYSGQVAPA---DDSSIAPSR---PRSY-SNYC-PPSLLEVHFIPKDHLFSDVILKS
Ptpu HPXA-2014466            -----------------------------------------------------------S
Mecr NRWZ-2048038            ------------------------------------------------------------
Paly YFGP-2105141            ------------------------------------------------------------
Pe sp. PIUF-2010895          ------------------------------------------------------------
Pene JHFI-2109909            ------------------------------------------------------------
Rina WJLO-2009253            TRNEYLERSP-----PQNFVPEGE---LRVS-AST----AHSEIRLKLKDSDFGEMTE--
Coco ILBQ-2045467            -RNGYLQRIV-----RQSSATEGA---FRIS-KSY----ALSHVPAQLKDSDFRDTL---
Mapo Mp6g09180.1             -RTGYHDQIV-----RQSSIIEGD---FRKS-TAY----ALPDVRVNLKGSSIRKSL---
Mapa HMHL-2053619            -RTGYHDQIV-----RQSPTIEGD---FRKS-TSY----ALPDVRVNLKGSSIRKTL---
Lucr TXVB-2075053            -RTGY---------IRNSPITASE---LRIS-NSY----AAPGAPASLKDSISQGTF---
Spte HERT-2044039            -REGYLEQIVH-----ESPIAAAREFPPSSS-VSY--TLQLEKLGVKFEDS---------
Blpu SRR8202193_DN28790      -RGGYVEKGV-----ADEPITVSE---LRYS-NSH----ALPEMYSRRKDSIFMEKS---
Hamn SRR8202204_DN3233       -TLGKGHQIGFLT-VPDKSIVSSD---ARAA-NFD--SSRHPSSFSKGRIQVFQRLSSF-
Trla SRR8202205_DN12243      -RKVNRGRIESGSPVTGKGLGSTAGRDLHSP-NSD----ALQRTVSSQKGRVFNLLNS--

                                                                                            180
Cafi RTMU-2183659            -SHSKPSGL---LSFLRRFV---------AELCRTSPV-----FDSWPAEKIMVQ-----
Scne IRBN-2167015            -GSHLKSRL---LNVLQRFT---------GQFWRTTI------PGSRPLEGSHDH--GKQ
Baba OFTV-2024263            -GSRLKSGL---LNVLQRFI---------AKFW-TVM------PGSRPLEGSHDH--IQQ
Odpr YBQN-2008974            ------------------------------------------------------------
Batr WZYK-2090729            -GSHSRSGL---WSTLRKFI---------DQFFRATI------LDSCSNEGLSDQ--YNG
Sc sp. LGOW-2014377          -------------------------------------------VNSWTVEVSQDY-PHRQ
Fror SRR8202176_DN2013       ------RGL---QGLVRKTFG-SGFDKHQTRMRIGLLEISRLIGPRRPFEVSPDH----Q
Raja SRR8202175_DN137879     -HSVSMGGL---QNLVRRFI-----NARQVQFEIDEHIIS-WEGATLPVRS-------HQ
Pona KRUQ-2018380            QSYFSITGL---QNLVRRFIA-AAIATHQIQFGIEAR------MNSLPAEASHDD--DRR
Popi UUHD-2013991            QSYLS--GI---RNLVRRFIA-AAIATHQIQFGIEAS------MNSLPAEASHDK--DHR
Ptpu HPXA-2014466            HAYYSRTGL---WNLVRRFIV---DVITKHQFYSAAF------INSWPVEALHDQYHYRH
Mecr NRWZ-2048038            ------------------------------------------------------------
Paly YFGP-2105141            ---------------------------------GEA--------NS-PAE----------
Pe sp. PIUF-2010895          ------------------------------------------------------------
Pene JHFI-2109909            ---------------MQKFI---ASVMKRVEFRKEL--------ISLPGGS-----QVRL
Rina WJLO-2009253            ---HADAG----VEVVPEFL---DTATGRTQFHGET--------NSPAAEDENG------
Coco ILBQ-2045467            ---HADGE----GDLVPKFL----DATGHNQFRYEA--------NSQTEE----------
Mapo Mp6g09180.1             ---HADSG----RSLVFNFL----DDIGPIQVGPEA--------ISRTEE----------
Mapa HMHL-2053619            ---HADSG----RSLGSNFL----DDMGPNQIGPEA--------ISRTEE----------
Lucr TXVB-2075053            ---HAAAGAIRGRNLVAKFL----DVMIQPQFGYGA--------NGSPAL----------
Spte HERT-2044039            ------GGL---QSATGKNVASLSIVMRQVQFHLGVH------GGVPTEE----------
Blpu SRR8202193_DN28790      ---LATTG----EILVRKIF---SAVISQTQFSFEA--------DS-PGE----------
Hamn SRR8202204_DN3233       -FKSEKTAL---ENLIGTIL-------KGIWFGGAA--------AQSTDEEKNEIC----
Trla SRR8202205_DN12243      ---YLSSE----KNLVESFI----------------GLI--INGTKSPDDTEVEK----G
```

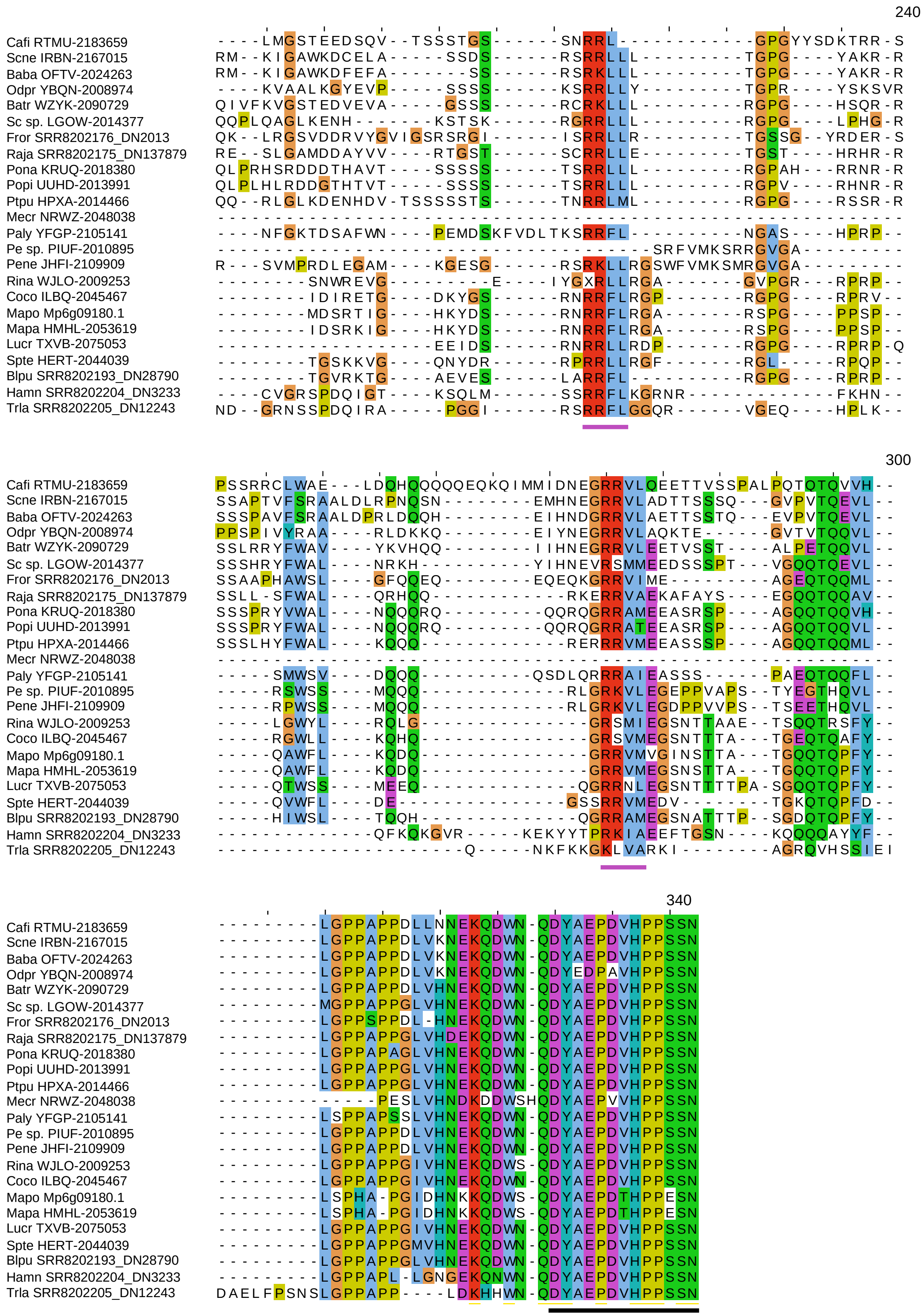

Supplementary Figure 1. Alignment of presumptive precursor amino acid sequences of the liverwort RGF-likes. Amino acids are color coded according to the chemical properties. Black and magenta bars below the alignment mark the predicted mature RGF peptide and potential proteolytic processing sites, respectively. Sequence titles are preceded by the abbreviated species name consisting of the first two letters of the generic and specific names.

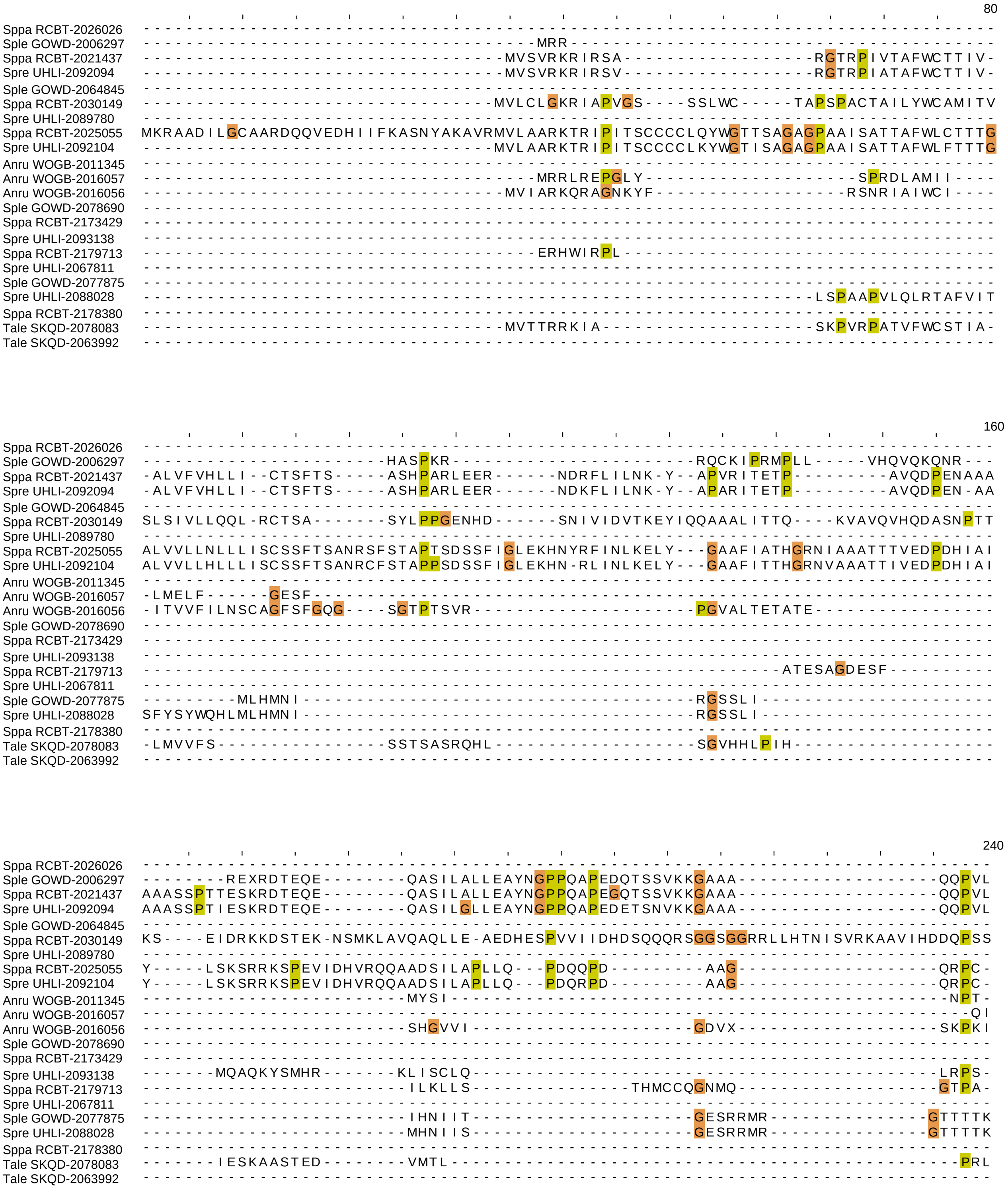

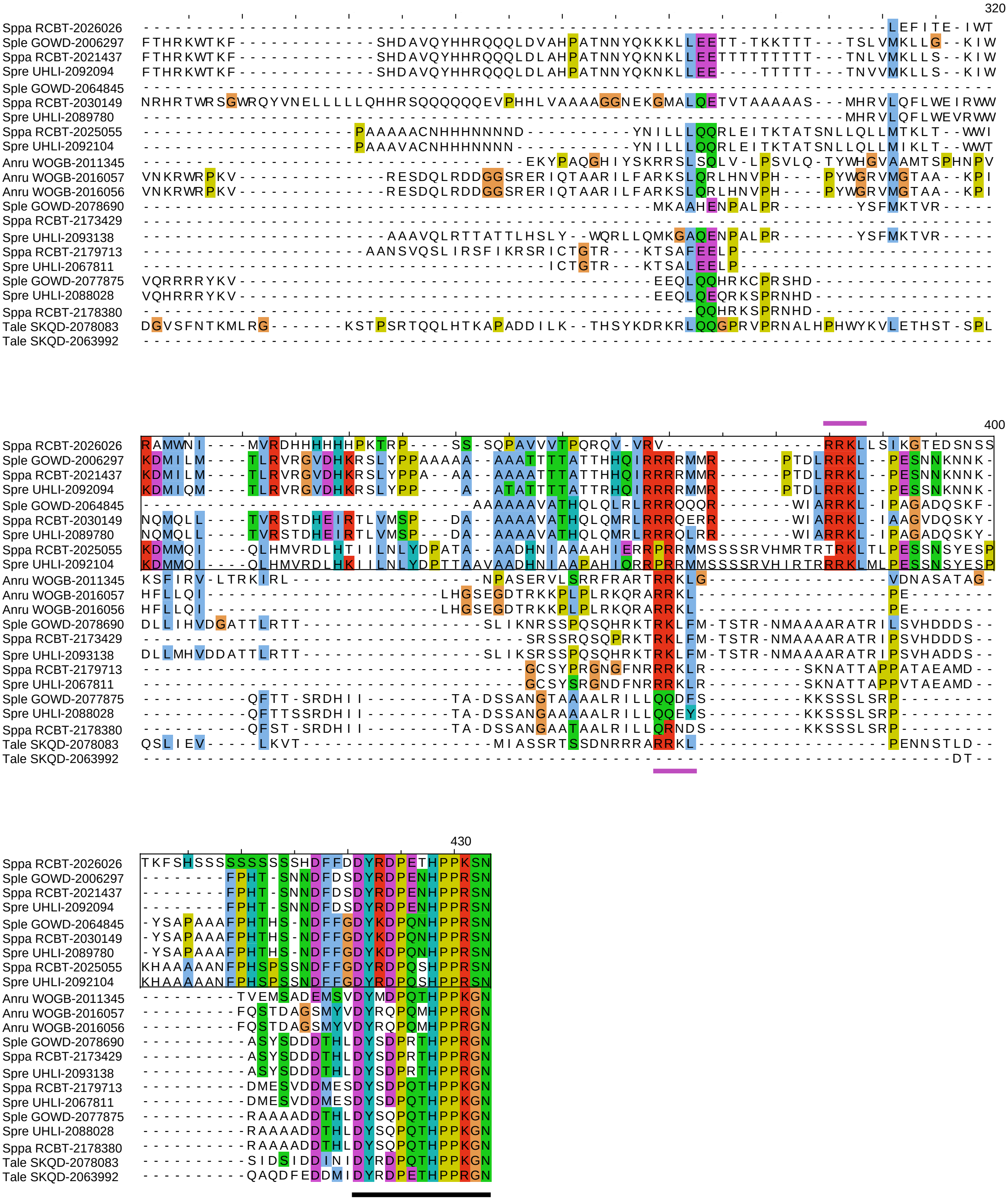

Supplementary Figure 2. Alignment of presumptive precursor amino acid sequences of the moss RGF-likes. In the last two rows, the top nine sequences are grouped to highlight characteristic residues. Amino acids are color coded according to the chemical properties. Black and magenta bars above and below the alignment mark the predicted mature RGF peptide and potential proteolytic processing sites, respectively. Sequence titles are preceded by the abbreviated species name consisting of the first two letters of the generic and specific names.

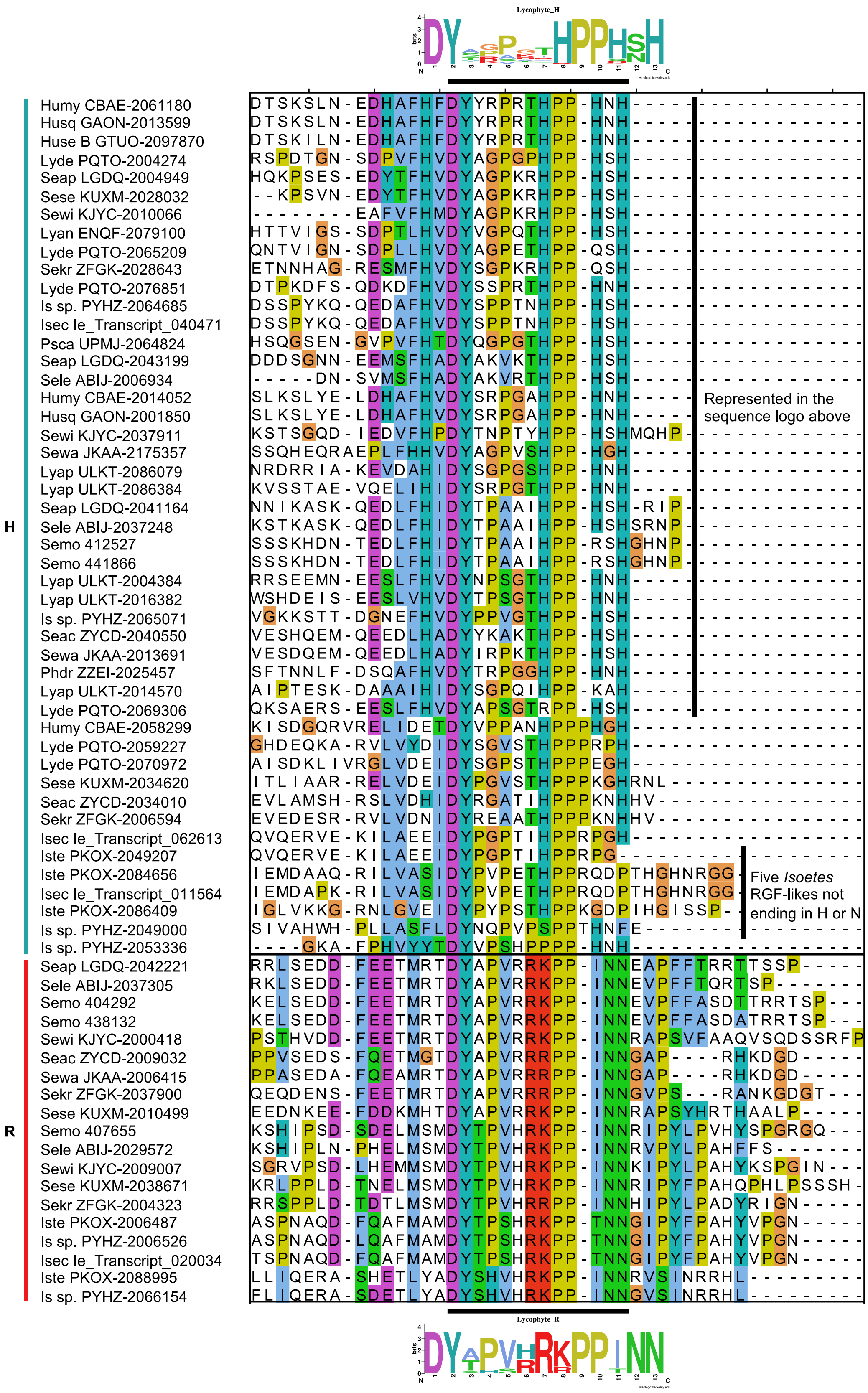

Supplementary Figure 3. Alignment of partial, C-terminal amino acid sequences of the lycophyte RGF-likes. Amino acids are color coded according to the chemical properties. Blue and red vertical lines beside the alignment mark H- and R-type RGF-like sequences, respectively. Above and below the alignment, thick black bars and sequence logos denote the positions and amino acid conservation of the predicted mature RGF peptides of the two subclasses. Sequence titles are preceded by the abbreviated species name consisting of the first two letters of the generic and specific names.

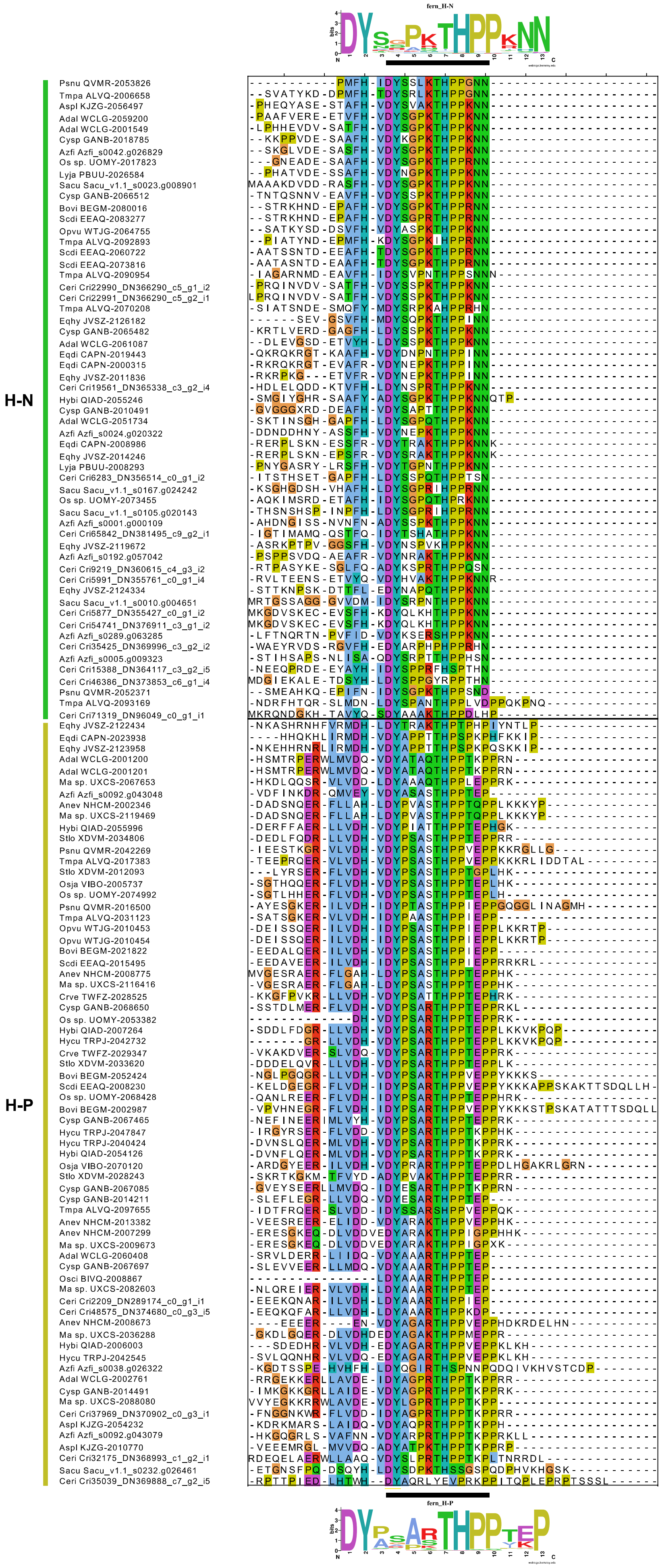
fern_H-N
H-N
H-P
fern_H-P

Supplementary Figure 4. Alignment of partial, C-terminal amino acid sequences of the fern RGF-likes. Amino acids are color coded according to the chemical properties. Green and yellow vertical lines beside the alignment mark N- and N-ending RGF-like sequences, respectively. Above and below the alignment, thick black bars and sequence logos denote the positions and amino acid conservation of the predicted mature RGF peptides of the two subclasses. Sequence titles are preceded by the abbreviated species name consisting of the first two letters of the generic and specific names.

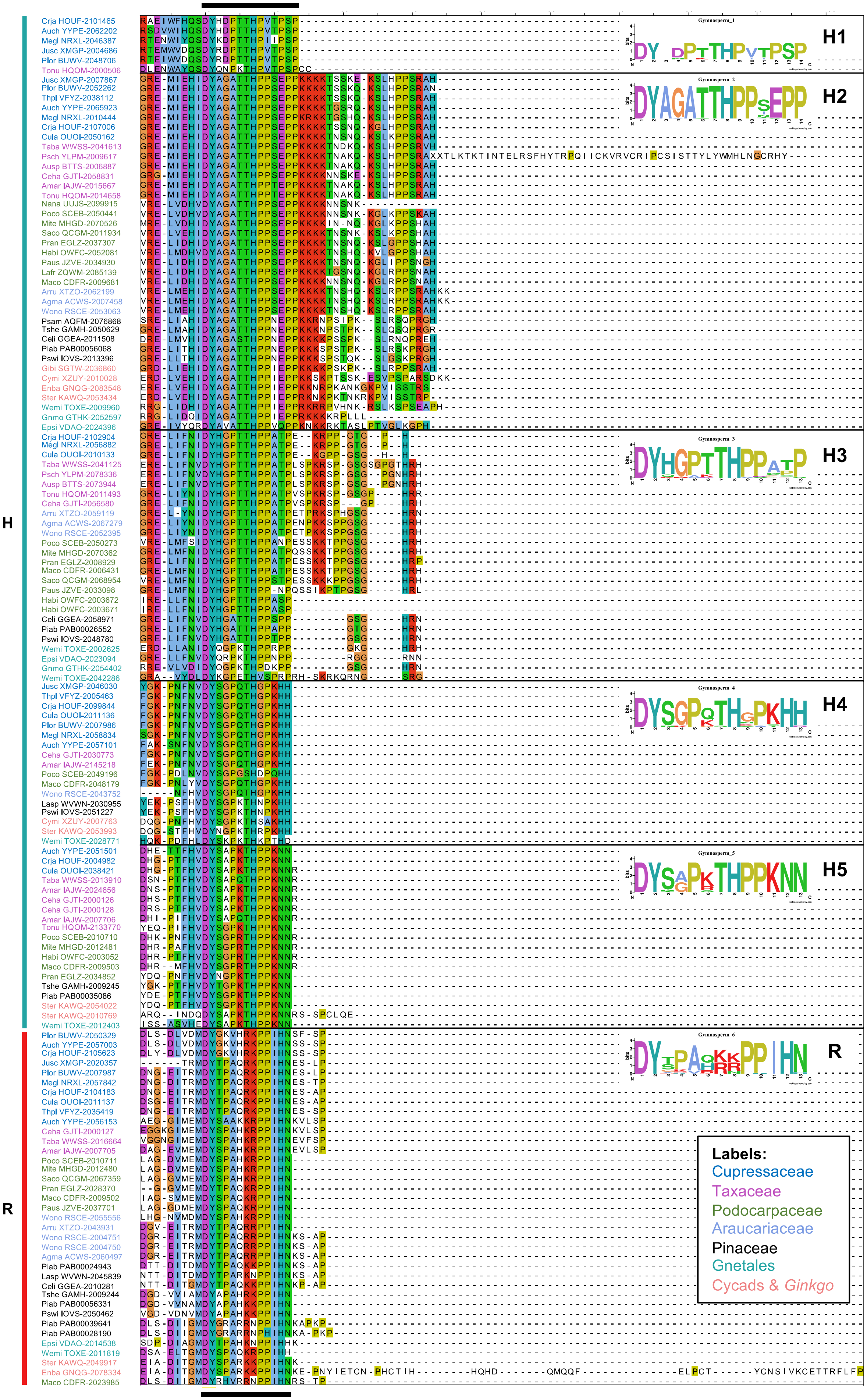
H1
H2
H3
H4
H5
R
H
Labels:
Cupressaceae
Taxaceae
Podocarpaceae
Araucariaceae
Pinaceae
Gnetales
Cycads & *Ginkgo*

Supplementary Figure 5. Alignment of partial, C-terminal amino acid sequences of the gymnosperm RGF-likes. Amino acids are color coded according to the chemical properties. Thick black bars above and below the alignment denote the positions of the predicted mature RGF peptides. Blue and red vertical lines beside the alignment mark H- and R-type RGF-like sequences, respectively. Group H is further classified into 5 subgroups based on the characteristic residues, and the sequence logo is shown for each subgroup at the upper right. For the subgroups H1 and H2, 14 amino-acid sequence logos are shown. Sequence titles are preceded by the abbreviated species name consisting of the first two letters of the generic and specific names. The same taxonomic color code is used to label sequence titles as in Figure 6.

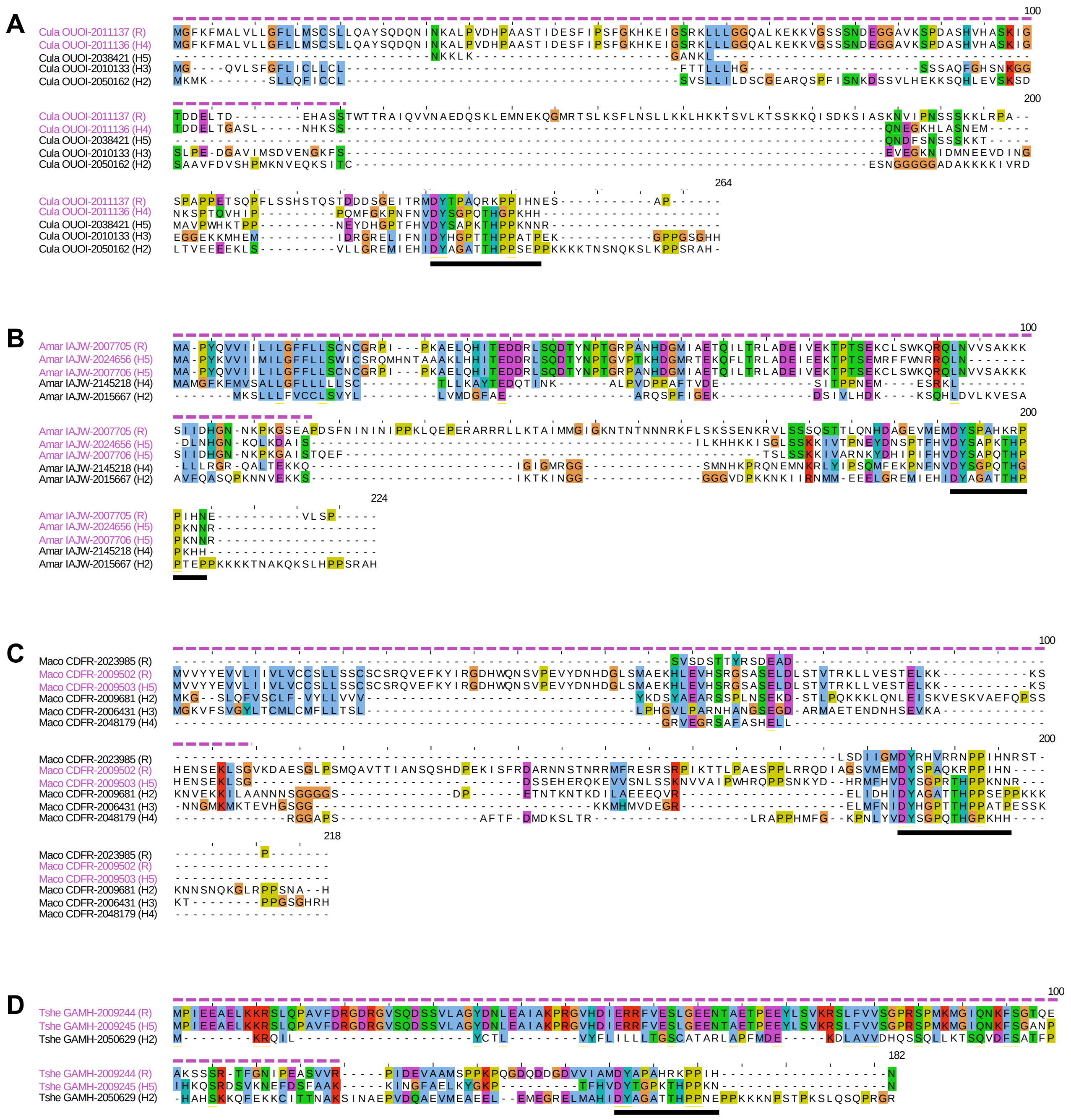

Supplementary Figure 6. Alignment of gymnosperm RGF-like sequences. Alignment of RGF-like precursor sequences from *Cunninghamia lanceolata* **(A)**, *Amentotaxus argotaenia* **(B)**, *Manoao colensoi* **(C)**, and *Tsuga heterophylla* **(D)** are shown. Amino acids are color coded according to the chemical properties. Sequence titles in magenta indicate that these precursors show N-terminal sequence similarities, the regions of which are indicated by dotted magenta bars above the alignment. In parentheses shown are the sequence classes described in Supplementary Figure 5. Thick black bars below the alignment mark the positions of the predicted mature RGF peptides.

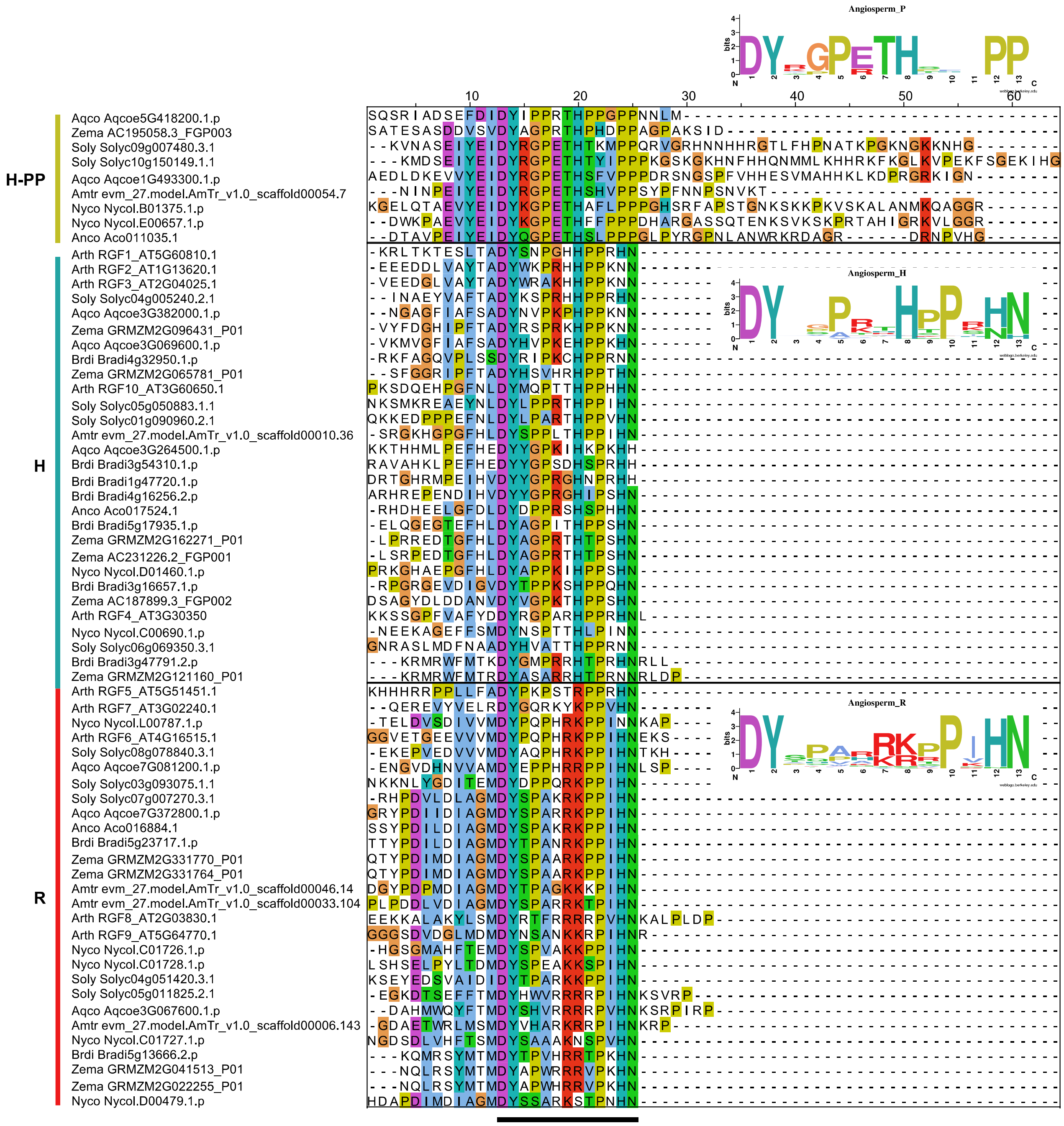

Supplementary Figure 7. Alignment of partial, C-terminal amino acid sequences of the angiosperm RGF-likes. Amino acids are color coded according to the chemical properties. A thick black bar below the alignment denotes the position of the predicted mature RGF peptide. Yellow, blue, and red vertical lines beside the alignment mark three groups, H-PP, H, and R-type, respectively. Sequence logos show the amino acid conservation of each group. Sequence titles are preceded by the abbreviated species name consisting of the first two letters of the generic and specific names.
