# Supplementary material for: The RGF/GLV/CLEL Family of Short Peptides Evolved Through Lineage-Specific Losses and Diversification and Yet Conserves Its Signaling Role Between Vascular Plants and Bryophytes
Source: Front Plant Sci. 2021 Jul 20;12:703012. doi: 10.3389/fpls.2021.703012 (PMC8329595; doi:10.3389/fpls.2021.703012)
Supplement: Supplementary file 4 [file Table_1.DOCX]

**Supplementary Table 1. Primers used in this study.**

| Name | Sequence (5’ to 3’) | Note |
| --- | --- | --- |
| CF273_AtRGF1-F1 | AGAGGACACGCTCGAGATGGTGTCCATAAGGGTTATTTGCTATC | In-Fusion cloning into pART7 |
| CF274_AtRGF1-R1 | GGACTCTAGAGGATCCTTAGTTATGCCTAGGAGGATGATGACC | In-Fusion cloning into pART7 |
| CF271_AtRGF6-F1 | AGAGGACACGCTCGAGATGTCTTGCTCTTTGAGGAGTGGACTCG | In-Fusion cloning into pART7 |
| CF272_AtRGF6-R1 | GGACTCTAGAGGATCCTTAAGACTTCTCGTTGTGGATCGGAGGC | In-Fusion cloning into pART7 |
| CF269_MpRGF-F11 | AGAGGACACGCTCGAGATGTTTAGATGCAGGTCAGAATGGG | In-Fusion cloning into pART7 |
| CF270_MpRGF-R6 | GGACTCTAGAGGATCCTCAGTTGCTTTCTGGAGGGTGTG | In-Fusion cloning into pART7 |
| CF277_MpRGF_mutagenesis-F1 | CCAGGATACGCACCCTCCAGAAAGCAACTGAG | Mutagenesis by inverse PCR |
| CF278_MpRGF_mutagenesis-R1 | GGGTGCGTATCCTGGCTCCAGTCTTGCTTC | Mutagenesis by inverse PCR |
| CF88_MpRGF-F7 | TCAGTCGACTGGATCATGTTTAGATGCAGGTCAGAATGGG | In-Fusion cloning into pENTR |
| CF89_MpRGF-R3 | GTCTAGATATCTCGATCAGTTGCTTTCTGGAGGGTGTGTATC | In-Fusion cloning into pENTR |
